# Characteristics and Regulating Roles of Wheat TaHsfA2-13 in Abiotic Stresses

**DOI:** 10.3389/fpls.2022.922561

**Published:** 2022-06-27

**Authors:** Xiangzhao Meng, Baihui Zhao, Mingyue Li, Ran Liu, Qianqian Ren, Guoliang Li, Xiulin Guo

**Affiliations:** ^1^Institute of Biotechnology and Food Science, Hebei Academy of Agriculture and Forestry Sciences/Plant Genetic Engineering Center of Hebei Province, Shijiazhuang, China; ^2^College of Life Sciences, Hebei Normal University, Shijiazhuang, China; ^3^College of Landscape and Ecological Engineering, Hebei University of Engineering, Handan, China

**Keywords:** wheat, Hsf, *TaHsfA2-13*, abiotic stresses, transgenic, *Arabidopsis*

## Abstract

Heat shock transcription factor (Hsf) exists widely in eukaryotes and responds to various abiotic stresses by regulating the expression of downstream transcription factors, functional enzymes, and molecular chaperones. In this study, *TaHsfA2-13*, a heat shock transcription factor belonging to A2 subclass, was cloned from wheat (*Triticum aestivum*) and its function was analyzed. *TaHsfA2-13* encodes a protein containing 368 amino acids and has the basic characteristics of Hsfs. Multiple sequence alignment analysis showed that TaHsfA2-13 protein had the highest similarity with TdHsfA2c-like protein from *Triticum dicoccoides*, which reached 100%. The analysis of tissue expression characteristics revealed that *TaHsfA2-13* was highly expressed in root, shoot, and leaf during the seedling stage of wheat. The expression of *TaHsfA2-13* could be upregulated by heat stress, low temperature, H_2_O_2_, mannitol, salinity and multiple phytohormones. The TaHsfA2-13 protein was located in the nucleus under the normal growth conditions and showed a transcriptional activation activity in yeast. Further studies found that overexpression of *TaHsfA2-13* in *Arabidopsis thaliana* Col-0 or *athsfa2* mutant results in improved tolerance to heat stress, H_2_O_2_, SA and mannitol by regulating the expression of multiple heat shock protein (*Hsp*) genes. In summary, our study identified *TaHsfA2-13* from wheat, revealed its regulatory function in varieties of abiotic stresses, and will provide a new target gene to improve stress tolerance for wheat breeding.

## Introduction

Abiotic stresses such as drought, high salt, and high temperature can affect the survival, productivity, and geographical distribution of many important crops worldwidely (Gong et al., 2020). There seem to be significant differences among the various stresses, while they also show overlapping responses. For example, osmotic stress caused by drought and salinity is an important environmental threat, limiting plant growth and agricultural productivity (Chen et al., 2020). Oxidative stress is an integral component of various stress conditions, including excessive light, cold, heat, drought, and this fact largely determines the substantial overlap in plant physiological and molecular responses to various environmental challenges (Savchenko and Tikhonov, 2021). In the process of fighting against these adverse environmental factors, plants have evolved a complex and elaborate regulatory network (Guo et al., 2016). With the development of molecular techniques such as large-scale transcriptome analysis, a large number of abiotic stress associated genes have been discovered in plants (Nakashima et al., 2009; Duan et al., 2019). Genes encoding transcription factors are downstream components of signal transduction and play indispensable roles in the regulation of multiple abiotic stresses. About 7% of the coding sequences in plant genomes are transcription factors, many of which typically belong to large gene families, such as the Hsf family (Baniwal et al., 2004; Udvardi et al., 2007). They are excellent candidates for crop improvement (Baillo et al., 2019). In contrast to the few Hsfs in yeast and mammals, the plant Hsf families contain a large number of Hsf members, forming a complex plant-specific superfamily that is widespread in many species (Guo et al., 2016). There are 21 *Hsfs* in *Arabidopsis*, 24 in tomato, 25 in pepper, 41 in bamboo, 52 in soybean, and 82 in wheat (Scharf et al., 2012; Fragkostefanakis et al., 2015; Guo et al., 2015; Duan et al., 2019; Huang et al., 2021). Compared to other plant species, the large number of *Hsfs* in wheat makes the corresponding research more complicated.

In general, plant Hsf proteins have conserved modular structures, including N-terminal DNA binding domain (DBD) characterized by a central helix-turn-helix motif, an oligomerization domain (OD) with a bipartite heptad pattern of hydrophobic amino acid residues (HR-A/B region), the C-terminal nuclear localization signal (NLS) and nuclear export signal (NES) for regulating Hsf proteins subcellular localization (Baniwal et al., 2004). Plant Hsf were classified into A, B, and C based on the sequence length between DBD and HR-A/B regions and the number of amino acid residues inserted into HR-A/B region (Guo et al., 2016; Liu et al., 2020). Among these three types of Hsf proteins, HsfA class members usually contain an activation domain at the C-terminal, characterized by aromatic, hydrophobic, and acidic amino acid residues (AHA), whereas HsfB and C lack the AHA motif and in many cases do not function as activator (Kotak et al., 2004). The different protein structures of HsfA, HsfB, and HsfC determine the different functions they perform. In *Arabidopsis*, HsfA1 and HsfA2 have been reported as positive regulators of plant responses to heat stress (HS), whereas two members of Class B Hsf, HsfB1, and HsfB2b, are active repressors of Hsfs and Hsps (Nishizawa et al., 2006; Charng et al., 2007; Ikeda et al., 2011; Liu et al., 2011; Nishizawa-Yokoi et al., 2011).

Current understanding about the individual functions for A2 subclass of Hsf in plant mainly comes from model species such as *Arabidopsis* and tomato (Heerklotz et al., 2001; Charng et al., 2007; Ogawa et al., 2007; Chan-Schaminet et al., 2009; Fragkostefanakis et al., 2016; Liu et al., 2019). The expression of *HsfA2* was not detected in control cell cultures, but was strongly upregulated after HS treatment, indicating an important regulatory role of *HsfA2* during HS in *Arabidopsis* (Nover et al., 2001). *HsfA2* transcript level was also strongly induced by HS in tomato (Scharf et al., 1998). In addition, the expression of *HsfA2* in tomato anther is higher than that in other flower tissues, and can be further induced under both short and prolonged HS conditions, similar to its expression in leaves (Giorno et al., 2010). Recent studies have found that HsfA2, as a central regulatory factor, is involved in the transgenerational memory to high temperature in plants through complex epigenetic regulation such as histone demethylation (Liu et al., 2019). In addition to heat stress, many other abiotic stresses such as cold, salt, and drought, as well as phytohormones such as abscisic acid (ABA), salicylic acid (SA), jasmonic acid (JA), and ethylene have also been shown to induce the expression of *HsfA2* in plants (Nishizawa et al., 2006; Zhang et al., 2015; Duan et al., 2019). As an important regulatory factor of HS, the *Arabidopsis* HsfA2 is involved in other abiotic stress, such as salt stress, osmotic stress and anoxia stress (Charng et al., 2007; Banti et al., 2010). Coincidentally, overexpression of *OsHsfA2e* in *Arabidopsis* also increased tolerance to high-salinity stress (Yokotani et al., 2008). Additionally, transgenic *Arabidopsis* plants overexpressing *TaHsfA2d* showed considerable tolerance to salinity and drought stresses (Chauhan et al., 2013). More recently, *TaHsfA2e-5D* has been shown to positively regulate heat and drought stress, at least in part by promoting the expression of stress-responsive genes (Bi et al., 2022). It seems that plant Hsfs may act as the intersection of multiple signaling pathways, on the one hand participating in the regulation of abiotic signaling pathways and on the other hand mediating phytohormone signaling (Guo et al., 2016). Indeed, our previous studies have shown that many Hsfs, such as the A2 subclass of Hsfs, are responsive to many abiotic stresses and phytohormones (Duan et al., 2019).

Although basic biological information about *Hsfs* in wheat is well known, fewer *Hsfs* have been cloned and identified from wheat than other crops, and the function of most Hsfs are unclear. Our previous studies have demonstrated the expression of *TaHsfA2-13* was induced by various abiotic stresses (Duan et al., 2019). In this study, the biological functions and potential mechanisms of wheat TaHsfA2-13 in plant tolerance to multiple abiotic stresses will be further revealed.

## Materials and Methods

### Plant Materials and Growth Conditions

Cang6005, a semi-winter heat-tolerant wheat variety, was used for gene cloning and expression analysis. After surface disinfection with 0.1% HgCl_2_, wheat seeds were cleaned with sterile water and soaked at room temperature for 12 h. When the germ grew to 1–2 cm, seedlings were transplanted and cultivated in Hoagland nutrient solution under day/night conditions of 16 h/8 h (70–100 μmol m^−2^ S^−1^), 25°C, and 50–60% relative humidity. The stress treatments were performed as described by previous studies (Duan et al., 2019; Liu et al., 2020; Bi et al., 2022). Seedlings at the two-leaf and one-heart stage were treated with high temperature (37°C), low temperature (4°C), H_2_O_2_ (10 mmol L^−1^), mannitol (15% W/V), NaCl (0.2 mol L^−1^), MeJA (0.1 mmol L^−1^), ABA (0.2 mmol L^−1^) and SA (0.8 mmol L^−1^) for different time, respectively. The second leaves were collected in each treatment, and then immediately frozen in liquid nitrogen for RNA extraction.

### Isolation of Total RNA and Gene Cloning

Total RNA of plants was extracted using the RNArose Reagent Systems Kit (Huashun Bioengineering Co., LTD., Shanghai, China), according to the manufacturer’s instructions. RNA was further purified through the PrimeScript^™^ RT Reagent Kit with gDNA Eraser (TaKaRa, Dalian) to remove genomic DNA contamination. Then, 2 μg of purified RNA was used as template to synthesize first-strand cDNA. The specific primers were designed using DNAMAN 8.0^1^ software to amplify the *TaHsfA2-13* sequence. The PCR amplified products were constructed to pEasy-Blunt cloning vectors (pEasy-Blunt Simple Cloning Kit, TransGen Biotech) and sequenced by Shanghai Shenggong Bioengineering Technology Co., LTD. Primer sequence information is presented in Supplementary Table S1.

### Multiple Sequence Alignment Analysis

The protein sequences used for multiple sequence alignment analysis were downloaded from NCBI website.^2^ GenBank accession numbers of the proteins are as follows: TaHsfA2-13 (*T. aestivum*, MT 680017), TdHsfA2c-like (*T. dicoccoides*, XP_037425119), AetHsfA2c (*Aegilops tauschii*, XP_020147337), BdHsfA2c (*Brachypodium distachyon*, XP_010234740), ZmHsf30 (*Zea mays*, NP_001147128), AtHsfA2 (*A. thaliana*, NP_001324815; Charng et al., 2007). Multiple sequence alignment analysis was carried out by the DNAMAN 8.0 software.^3^

### Expression Analysis Using Quantitative RT-PCR

Real-time PCR was performed with SYBR Green PCR master mix (TaKaRa, Dalian) on a Bio-Rad CFX96 realtime PCR detector. According to the methods of Liu et al. (2020), *TaRP15* and *Atactin8* were selected as internal reference genes in wheat and *Arabidopsis*, respectively, to calculate the relative transcription levels of the interest genes. The primers used are listed in Supplemental Table S1. Three replicates were performed for each qRT-PCR reaction, and the results were reported as means and standard deviation. SPSS 19.0 software was used to analyze the significance.

### Subcellular Localization

The coding sequence of *TaHsfA2-13* without a stop codon was inserted into the pJIT1-hGFP vector through LR recombination reaction to generate TaHsfA2-13-GFP fusion protein. The vectors was transformed into *Agrobacterium* strain GV3101 and injected into *N. benthamiana* leaves with a needleless 1 ml syringe. The nucleus was stained with 4′,6-diamidino-2-phenylindole (DAPI, 1 mg ml^−1^) dye before imaging, fluorescence observation was performed under a fluorescence microscope (Zeiss META510).

### Transactivation Analysis

The full-length and truncated *TaHsfA2-13* were cloned into pGBKT7 and then introduced into yeast strain AH109, respectively. The transformants were grown on the SD/-Trp and SD/Trp-/His-/Ade-media for 3 days before observation. The transcriptional activation activities was evaluated according to their growth status and the activity of α-galactosidase.

### Yeast One-Hybrid Assay

For the construction of prey, the coding sequence of *TaHsfA2-13* was amplified from wheat cDNA and then cloned into pGADT7 vector. For bait construction, the promoters of five *AtHsps* were amplified from *Arabidopsis* genomic DNA and then cloned into pHIS2.1 vector according to the methods described by Liu et al. (2020). The bait and prey constructs were co-transformed into yeast strain Y187, and grown on the selective medium with 10 mM 3-aminotriazole (3-AT) or not.

### Genetic Transformation

To obtain the plants overexpressing *TaHsfA2-13* in *Arabidopsis*, the coding region of *TaHsfA2-13* was amplified by PCR and cloned into pCAMBIA1300. The recombinant plasmid was transformed into *Agrobacterium* strain GV3101. At the stage of flower-budding, *Arabidopsis* wild type (Col-0) and *athsfa2* mutants were transformed by classical floral dip method (Liu et al., 2020). The T-DNA insertion transgenic seeds were selected on MS-agar plates supplemented with 50 μg·μL^−1^ of hygromycin and confirmed by semi-quantitative RT-PCR. *Atactin8* gene was used for normalizing the gene expression in *Arabidopsis*.

### Stress Treatments

For heat stress treatment, sterilized seeds of wild-type (or *athsfa2*) and *Arabidopsis* homozygous transgenic lines were planted on MS agar plates. Seedlings of 5 days old were subjected to basal thermotolerance (45°C for 50 min, placed at 22°C for 8 days) and acquired thermotolerance treatment (37°C for 1 h, incubated at 22°C for 2 days, 46°C for 50 min, placed at 22°C for 8 days) according to the previous methods (Larkindale et al., 2005; Li et al., 2019). After 8 days, photography was taken and the rosette leaves of different lines were collected to determine chlorophyll content. For oxidative stress, osmotic stress and phytohormone treatments, sterilized wild-type and transgenic seeds were planted on MS agar plates supplemented with H_2_O_2_ (10 mmol l^−1^), mannitol (15% W/V), and SA (0.8 mmol l^−1^), respectively. After 2 weeks, photography was taken and the rosette leaves of different lines were collected to determine chlorophyll content. The chlorophyll contents were measured as described by Liu et al. (2020). In each experiment, 30 seedlings of each line were treated at least, and three independent experiments were conducted.

### Measurements of Physiological Indexes

The rosette leaves of *Arabidopsis* seedlings were harvested to measure the physiological indexes after seedlings of 5 days old were subjected to basal thermotolerance (45°C for 50 min, placed at 22°C for 8 days). The activities of superoxide dismutase (SOD) and peroxidase (POD) were analyzed using the nitrotetrazolium blue chloride reduction and guaiacol colorimetric method, respectively (Li et al., 2015). Osmotic potential was determined with a VIESCOR 5100C vapour pressure osmometer (Wesor Inc., Logan, UT, United States of America) as described by Liu et al. (2002).

## Results

### cDNA Sequence Amplification and Analysis of TaHsfA2-13 in Wheat

**Figure 1:**
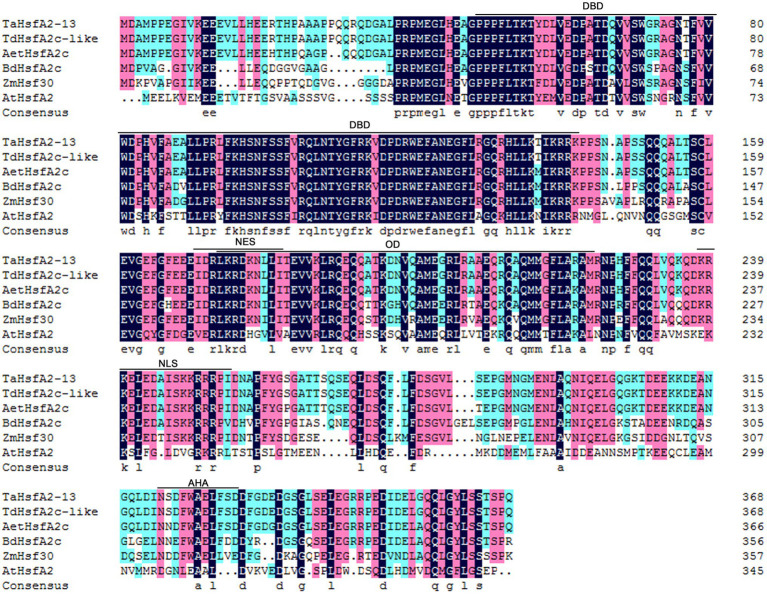
Sequence alignment of TaHsfA2-13 and other plant HsfA2 proteins. Multiple sequence alignment of amino acid sequences from *Aegilops tauschii* (AetHsfA2c), *Arabidopsis thaliana* (AtHsfA2), *Brachypodium distachyon* (BdHsfA2c), *Triticum aestivum* (TaHsfA2-13), *Triticum dicoccoides* (TdHsfA2c-like), and *Zea mays* (ZmHsf30). Conservative domains such as: DNA-binding domain (DBD), Oligomerization domain (OD), Nuclear localization signal (NLS), Nuclear export signal (NES) and Activator peptide motif (AHA) are marked with line segments.

Our previous RNA-Seq data showed that *TaHsfA2-13*, a Hsf of subclass A2, was strongly upregulated by heat stress, H_2_O_2_ and ABA, suggesting that this gene may be an important node in abiotic stress and phytohormones (Duan et al., 2019). To further clarify the biological function of TaHsfA2-13, we obtained the coding sequence of it from heat-treated wheat leaves, which encoding 369 amino acids. Analysis of amino acid sequence showed that TaHsfA2-13 had the basic characteristics similar to other Hsfs of class A, including DNA binding domain (DBD), oligomerization domain (OD), nuclear localization signal (NLS), nuclear export signal (NES) and activator peptide motif (AHA). Moreover, multiple sequence alignment analysis revealed that TaHsfA2-13 shared the highest similarity with TdHsfA2c-like from *T. dicoccoides* (Figure 1).

### Expression of *TaHsfA2-13* Was Induced by Various Abiotic Stresses and Phytohormones

**Figure 2:**
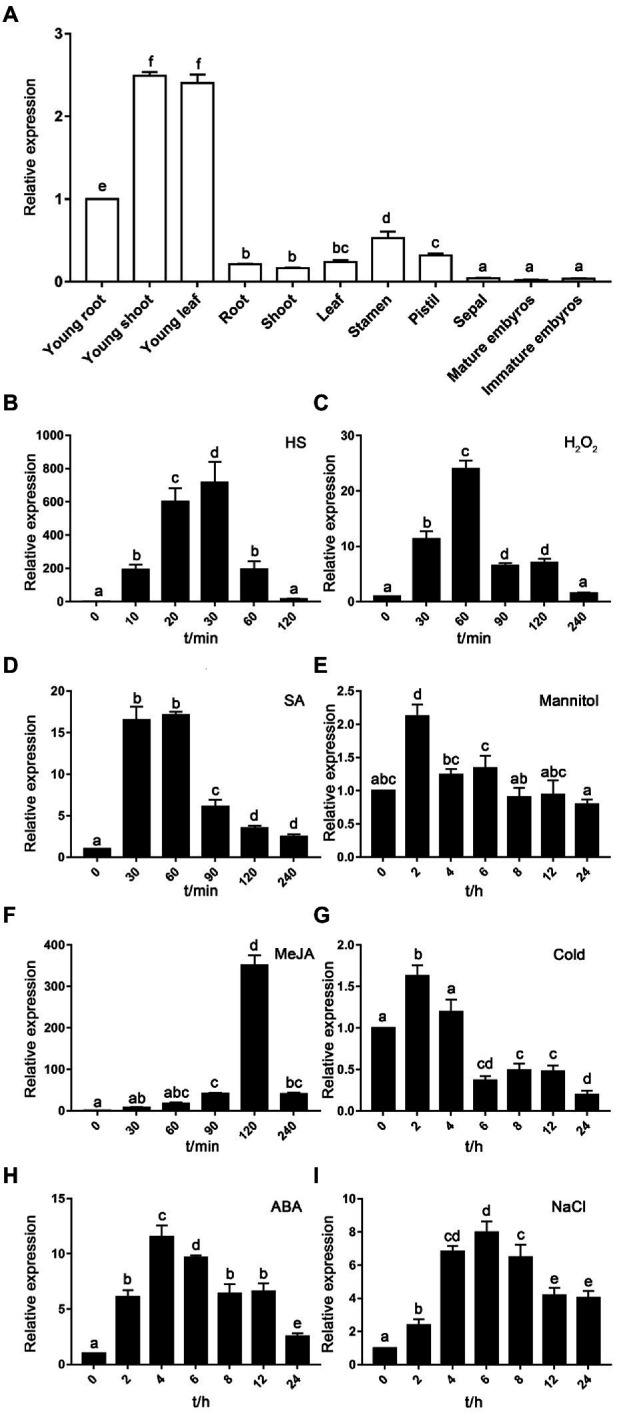
Expression analysis of *TaHsfA2-13*. **(A)** Tissue-specific expression patterns of *TaHsfA2-13*. The expression level in young root was set as 1. **(B–I)** Expression levels of *TaHsfA2-13* in response to abiotic stresses and phytohormones. The expression of untreated samples was set as 1. The wheat *TaRP15* gene was used as an internal control. All data represent means ± standard deviation (SD) of three biological replicates. Different lowercase letters above the bars denote significant differences at the *p* < 0.05 level.

In order to explore the expression patterns of *TaHsfA2-13* in wheat, qRT-PCR was used to detect the expression of *TaHsfA2-13* in different tissues at seedling and reproductive stages. Compared with the reproductive stage of wheat, *TaHsfA2-13* was more greatly expressed in seedling stage, and the expression level in young shoot or leaf was 2.5 fold higher than that in root under normal growth conditions. Specifically, the expression level of *TaHsfA2-13* in embryos was almost undetectable, suggesting that *TaHsfA2-13* may not be involved in embryonic development (Figure 2A).

Next, to confirm the induction of *TaHsfA2-13* gene expression under various abiotic stresses and phytohormones, we detected the transcript levels of *TaHsfA2-13* in wheat leaves which were treated with 37°C HS treatment, 4°C cold treatment, H_2_O_2_, mannitol, NaCl, MeJA, ABA and SA, respectively. It was found that the *TaHsfA2-13* gene was substantially induced by HS and its highest level was about 700 fold greater than the untreated control (Figure 2B). Under H_2_O_2_ treatment, the expression level of *TaHsfA2-13* was greatly increased and reached the peak at 60 min (Figure 2C). Following NaCl stress, *TaHsfA2-13* expression was induced at 2 h, peaked at 6 h, and then decreased slowly (Figure 2I). However, for mannitol and cold treatments, the responses of *TaHsfA2-13* were relatively weak, and its transcript levels slightly increased at 2 h (Figures 2E,G). Strikingly, the responses of *TaHsfA2-13* to phytohormones were extremely differently. SA treatment could rapidly induce expression of *TaHsfA2-13* to the peak within 30 min (Figure 2D). Compared with the SA treatment, the peak time of *TaHsfA2-13* transcription level reached under the MeJA and ABA treatments was delayed, which was 120 min and 2 h, respectively (Figures 2F,H).

### TaHsfA2-13 Is a Transcriptional Activator Localized to the Nucleus

**Figure 3:**
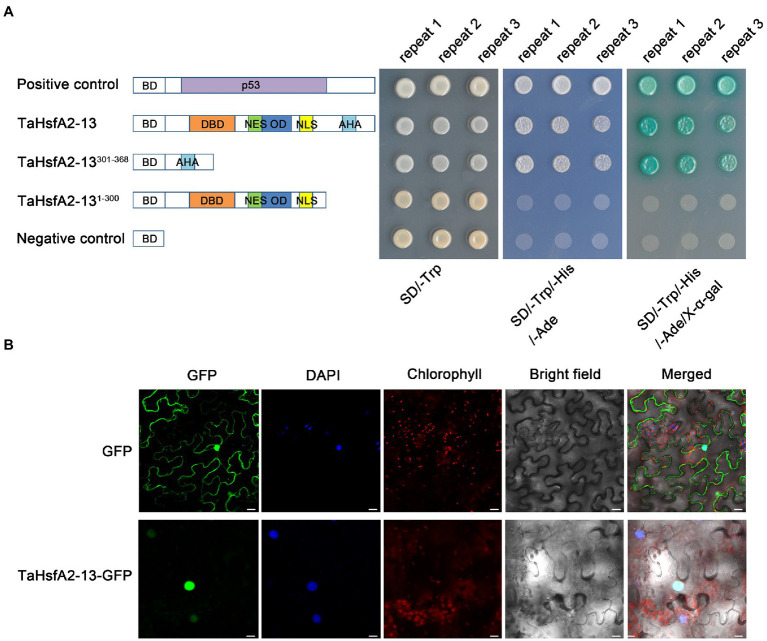
Transcriptional activity and subcellular localization analyses of TaHsfA2-13. **(A)** Transcriptional activation of TaHsfA2-13 in yeast. Schematic representation of the full-length TaHsfA2-13 and truncated protein constructs in the pGBKT7 vector. Fusion proteins of the GAL4 DNA-binding domain (BD) and full-length TaHsfA2-13 or truncated TaHsfA2-13 with (301–368 aa) or without (1–300 aa) AHA domain were expressed in the yeast strain AH109. Transformants were spotted onto SD/-Trp and SD/Trp-/His-/Ade-media. The plates were incubated at 30°C for 3 days. The pGBKT7 and pGBKT7-P53 vectors were used as negative and positive controls, respectively. **(B)** Subcellular localization of TaHsfA2-13 in tobacco leaf epidermal cells. Bars = 20 μm.

As shown in Figure 1, TaHsfA2-13 has an AHA domain at the C-terminal of its protein, suggesting that TaHsfA2-13 may be a transcriptional activator. To verify this, we analyzed the transcriptional activation activity of TaHsfA2-13 in yeast with the GAL4 yeast system. The yeast harboring pGBKT7-TaHsfA2-13 and pGBKT7-TaHsfA2-13^301-368^ grew well like positive controls, which carrying pGBKT7-P53 on the yeast medium (SD/Trp-/His−/Ade−/X-α-gal), while the yeast harboring pGBKT7-TaHsfA2-13^1-300^ and negative controls barely grew at all (Figure 3A). These results indicate that TaHsfA2-13 has a transcriptional activation activity in yeast.

Analysis of amino acid sequence revealed that TaHsfA2-13 possesses both NLS and NES (Figure 1), so the true localization of its protein is worth considering. The transient expression vector pJIT1-TaHsfA2-13-hGFP was constructed and transformed into tobacco leaf epidermal cells. The subcellular localization of the target protein was revealed by fluorescence microscope. Under normal circumstances, compared to the whole cell distribution of free GFP, the green fluorescence of the target protein is only distributed in the nucleus, which completely overlaps with the fluorescence of the nuclear specific dye DAPI. This indicates that TaHsfA2-13 is a nuclear localization transcription factor (Figure 3B).

### The Overexpression of *TaHsfA2-13* Enhanced the Tolerance of Transgenic *Arabidopsis* to Various Abiotic Stresses

**Figure 4:**
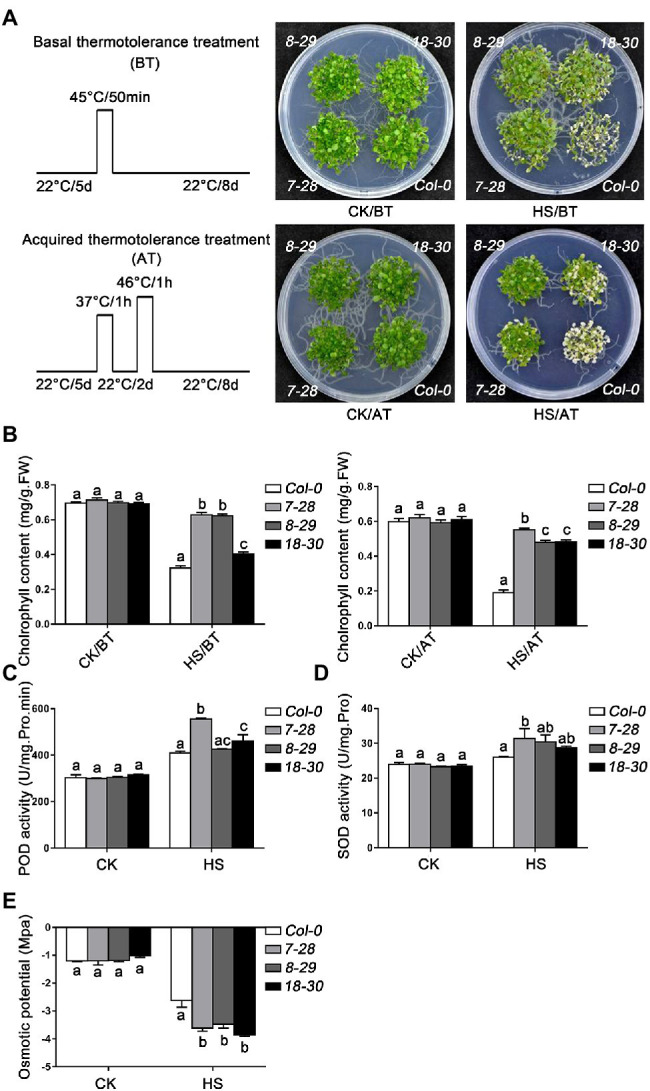
Thermotolerance assays of *TaHsfA2-13* overexpressing transgenic *Arabidopsis* lines. **(A)** The phenotypes of WT and transgenic seedlings after basal thermotolerance (BT, 45°C for 50 min, placed at 22°C for 8 days) and acquired thermotolerance (AT, 37°C for 1 h, incubated at 22°C for 2 days, 46°C for 1 h, placed at 22°C for 8 days) treatment. The plants were photographed 8 d after different HS. **(B)** Chlorophyll content of WT and transgenic seedlings treated with different HS. **(C**–**E)** Peroxidase (POD), superoxide dismutase (SOD) activities, and osmotic potential of WT and transgenic seedlings under HS treatment or not. For each experiment, at least 30 plants per line were used. Values are means ± SD from three independent measurements. Different lowercase letters above the bars denote significant differences at the *p* < 0.05 level.

To further explore the biological functions of TaHsfA2-13 in plants, transgenic plants overexpressing *TaHsfA2-13* in Col-0 (*7–28*, *8–29* and *18–30*) or *athsfa2* mutant (*6–11*, *10–16* and *11–12*) were obtained (Supplementary Figure S1). Firstly, the role of TaHsfA2-13 in thermotolerance was analyzed. As shown in Figure 4A; Supplementary Figure S2, under normal growth conditions, there was no significant growth difference between transgenic lines and WT (or *athsfa2*). However, under heat stress conditions, transgenic plants had fewer withered leaves and better growth than WT (or *athsfa2*). Consistent with this, the content of chlorophyll in transgenic plants was also significantly higher than that in WT (or *athsfa2*), indicating a less degree of injury in transgenic lines after HS treatment (Figure 4B). Meanwhile, we also detected the activities of POD, SOD and osmotic potential in transgenic lines and WT under HS treatment or not. The results showed that under normal growth conditions, there was no difference between the transgenic lines and WT. But, when subjected to HS treatment, the activities of POD and SOD were significantly higher in transgenic lines, and osmotic potential of the transgenic lines was significantly lower (Figures 4C–E).

**Figure 5:**
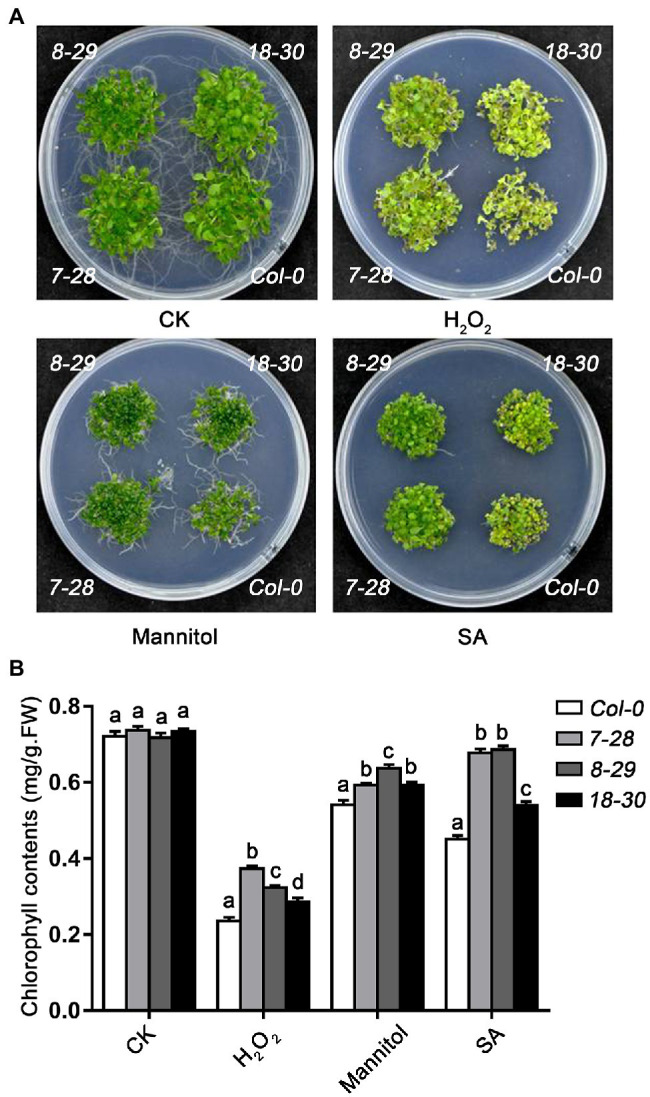
Effect of H_2_O_2_, salicylic acid (SA) and mannitol on WT, and transgenic plants. **(A)** Morphology and **(B)** Chlorophyll content of WT and transgenic seedlings after H_2_O_2_ (10 mmol L^−1^), mannitol (15% W/V), and SA (0.8 mmol L^−1^) treatment. The data was recorded after 2 weeks of germination on MS agar plates. For each experiment, at least 30 plants per line were used. Values are means ± SD from three independent measurements. Different lowercase letters above the bars denote significant differences at the *p* < 0.05 level.

Next, we also explore the role of *TaHsfA2-13* in oxidative stress, osmotic stress and response to phytohormone. As expected, following the H_2_O_2_, SA and mannitol treatments, the growth of transgenic seedlings was better than that of WT (or *athsfa2*), and the chlorophyll content of transgenic seedlings was higher, suggesting that TaHsfA2-13 could also enhance the resistance to oxidative stress, osmotic stress and toxicity of excess phytohormone other than improve the basic and acquired thermotolerance of transgenic plants (Figures 5A,B; Supplementary Figure S2).

### Expression Analysis of *Hsps* in the *TaHsfA2-13* Overexpressing Lines

**Figure 6:**
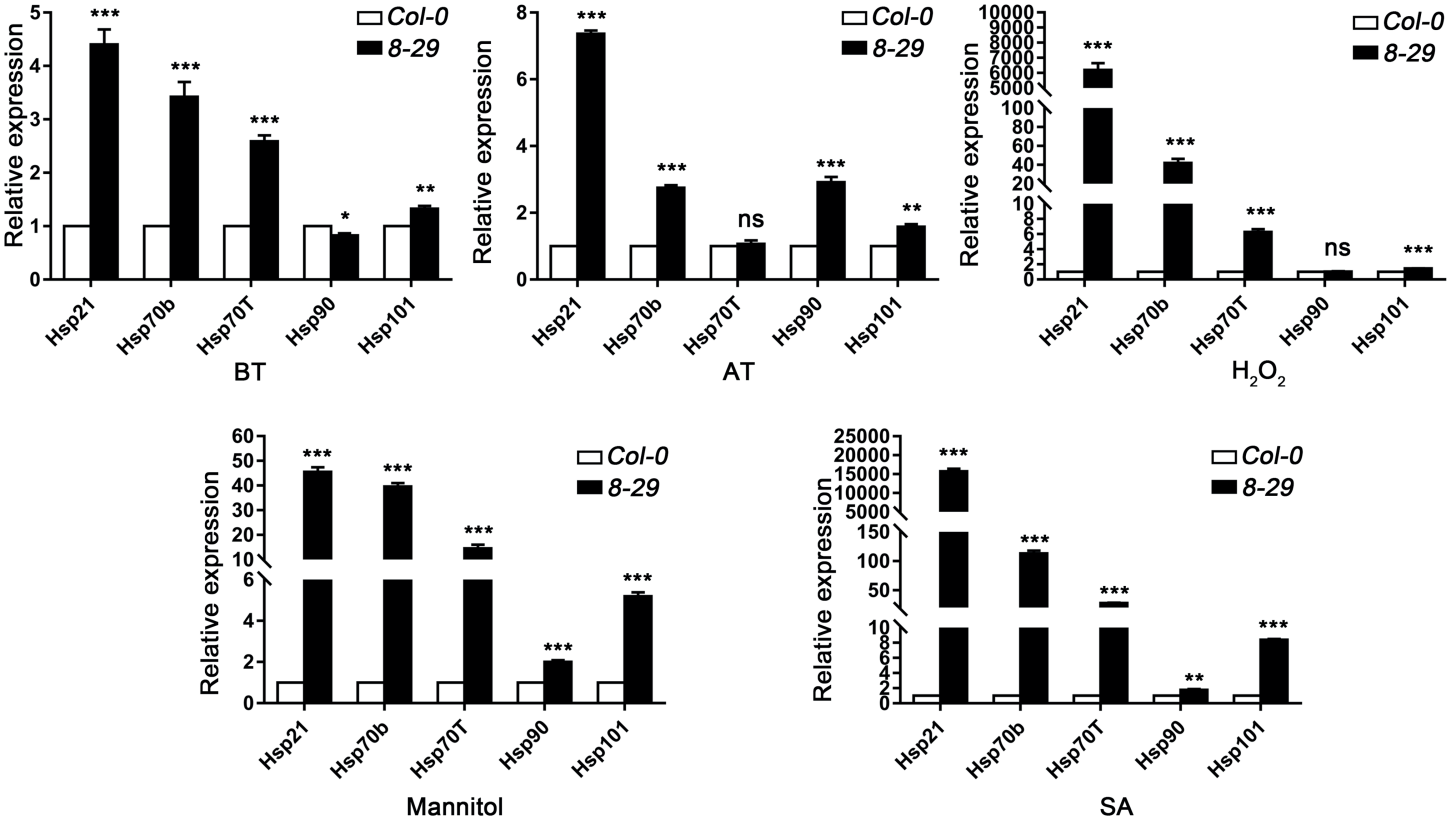
Expression analysis of *Hsp* genes in WT and transgenic lines after various stresses and phytohormones treatments. For analysis of *Hsp* genes transcription levels under heat stress, total RNAs were extracted from five-old day seedlings after treatment with slightly modified BT (45°C for 50 min) or AT (37°C for 1 h, incubated at 22°C for 2 days, 46°C for 1 h). For analysis of *Hsp* genes transcription levels under H_2_O_2_, SA and mannitol treatments, total RNAs were extracted from 2-week-old seedlings grown on MS plates with H_2_O_2_ (10 mmol L^−1^), mannitol (15% W/V), and SA (0.8 mmol L^−1^). The relative expression of *Hsp* genes were normalized to that of *Atactin8*, which was used as an internal control gene. All values are presented as the mean ± standard error of values from three independent experiments. Asterisks indicate significant differences compared to WT, and ns means no significant difference (^*^p < 0.05, ^**^p < 0.01, ^***^p < 0.005, Student’s *t*-test).

**Figure 7:**
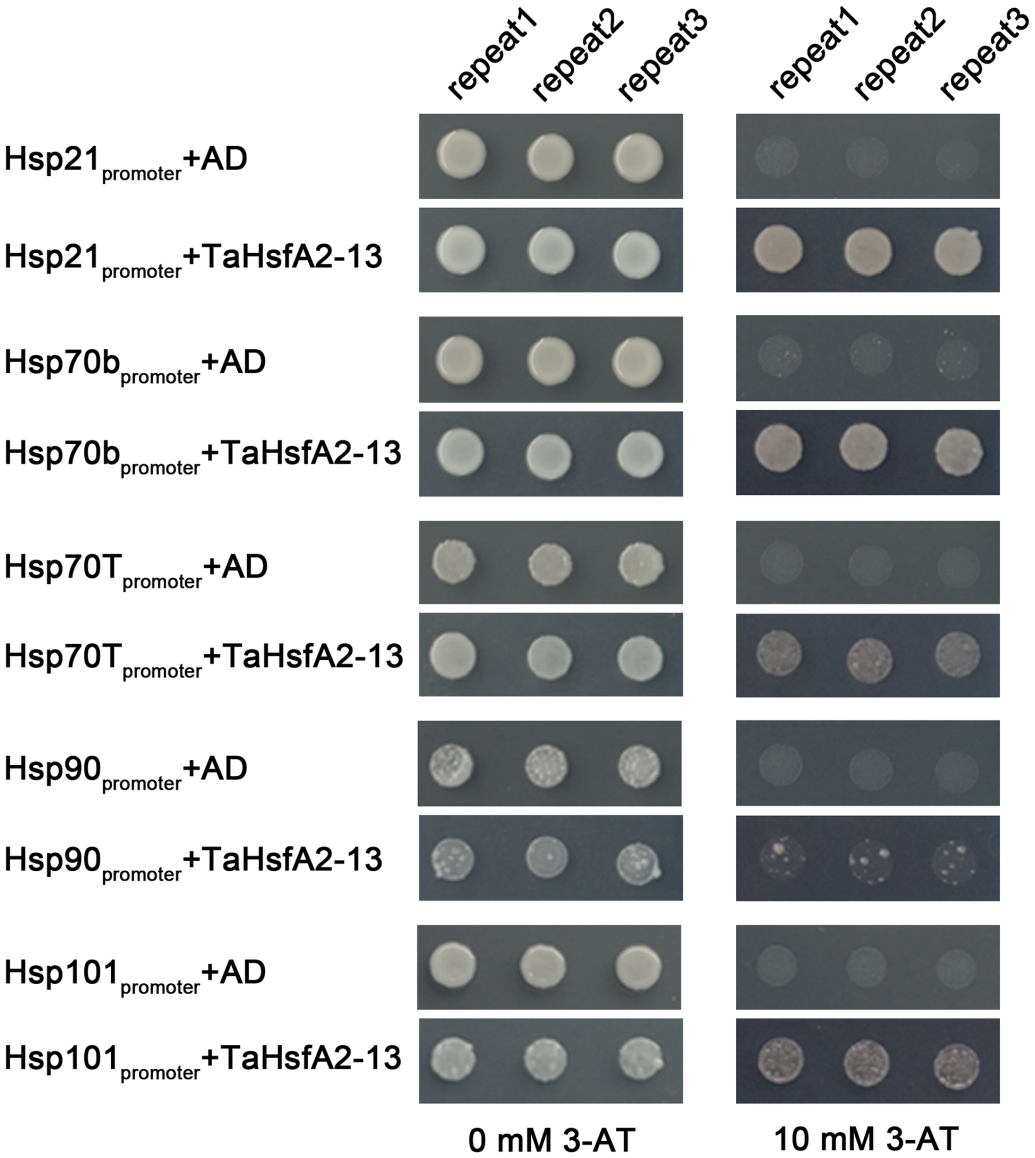
Growth of yeast cells transformed with the bait and prey constructs on SD-Trp-Leu-His supplemented with or without 10 mM 3-AT. The transformation with the empty vector pGADT7 was taken as a negative control. The experiments were repeated three times with the same results.

To determine the regulatory role of TaHsfA2-13 on *Hsps* in *Arabidopsis*, five *Hsp* genes, including *Hsp21*, *Hsp70b*, *Hsp70T*, *Hsp90*, *Hsp101*, which can be positively regulated by AtHsfA2 were selected in this study (Nishizawa et al., 2006; Ogawa et al., 2007). After mannitol and SA treatments, the expression levels of these *Hsp* genes were higher in the *TaHsfA2-13* overexpressing lines than those in WT. However, not all the expression levels of these *Hsp* genes were higher in the *TaHsfA2-13* overexpressing lines, especially after heat treatment and H_2_O_2_ treatment. During basic thermotolerance, the expression level of *Hsp90* in transgenic lines was significantly lower than that in WT. The transcript levels of *Hsp70T* and *Hsp90* in transgenic lines were not changed during acquired thermotolerance or H_2_O_2_ treatment compared with WT (Figure 6). Further experiments showed that TaHsfA2-13 could interact with promoter sequences of these *Hsps*, which containing HSE motifs (Figure 7). These results suggest that TaHsfA2-13 enhances the tolerance of transgenic lines to various abiotic stresses, probably mediates by activation of *Hsps*. Of course, more experiments are needed to explore the specific molecular mechanisms involved in regulating plant responses to various abiotic stresses.

## Discussion

Wheat is one of the important staple food crops, providing 21% of calories and 20% of protein globally (Lobell and Gourdji, 2012; Shiferaw et al., 2013). It is estimated that the demand for wheat will increase by 60% by 2050, but yields might go down by 29% because of environmental stresses (Manickavelu et al., 2012; Kulkarni et al., 2017). The main objective of agronomic research remains to improve crop productivity under various abiotic stresses (Puranik et al., 2012). Therefore, understanding the molecular mechanisms of plant responses to abiotic stresses is a prerequisite for improving plant stress tolerance and productivity (Guo et al., 2016). Hsfs are downstream components of signal transduction and play important roles in regulating the expression of many genes responding to various environmental stresses (Hrmova and Hussain, 2021). Despite there are large number of Hsfs in wheat, few Hsfs have been cloned and identified due to the complexity of the wheat genome. In this study, we cloned *TaHsfA2-13*, a member of the A2 subclass, from wheat. Analysis of amino acid sequence showed that TaHsfA2-13 possessed the basic properties of HsfAs (Figure 1). Unlike HsfBs and HsfCs, HsfAs tend to have the AHA domain, which also determines their transcriptional activation activity (Kotak et al., 2004). Indeed, TaHsfA2-13 was found to exhibit transcriptional activation activity in this study (Figure 3A). Similarly, in a recent study, the AHA motif was found to be important for the transcriptional activity of TaHsfA2e-5D (Bi et al., 2022). Although NLS and NES coexist, TaHsfA2-13 is normally located in the nucleus (Figures 1, 3B), which differs its orthologue from *Arabidopsis* (Evrard et al., 2013). Furthermore, the sequence of TaHsfA2-13 is highly similar to TdHsfA2c-like from diploid *T. dicoccoides*, indicating that TaHsfA2-13 is highly conserved during the polyploidization (Figure 1). In the A, B and D subgenomes of common bread wheat, approximately 60.1–61.3% of the genes have orthologues in diploid species (International Wheat Genome Sequencing, 2014). It seems that polyploidization has been shown to enhance abiotic stress tolerance in plants by aggregating stress resistance genes (Chao et al., 2013; Yang et al., 2014).

Although there is high sequence similarities among members of the A2 subclass, the expression pattern of each gene is quite different (Duan et al., 2019). In this study, gene expression profiles showed that *TaHsfA2-13* transcripts were differentially accumulated in all tested tissues, and the highest expression level was found in young roots, stems, and leaves, suggesting that TaHsfA2-13 might be involved in the growth of wheat seedlings (Figure 2A). However, previous study showed that subclass A2 members including A2b, A2c, and A2e were predominantly expressed in endosperm (Xue et al., 2014). *TaHsfA2-1*, also belonging to A2 subclass, was mainly expressed in mature leaves (Liu et al., 2020). These results indicate the functional diversity of A2 subclass members in wheat.

In recent years, there have been many reports about Hsfs regulating plant responses to various abiotic stresses and these transcription factors are not only involved in individual stress. For example, overexpression of *AtHsfA2* in *Arabidopsis* increases tolerance to heat, osmosis, and salt stress (Nishizawa et al., 2006). TaHsfA6f can enhance the tolerance of transgenic plants to high temperature, drought and salt stress in wheat (Xue et al., 2015; Bi et al., 2020). Like many Hsfs, TaHsfA2-13 not only improves the basic and acquired thermotolerance of transgenic plants, but also involve in response and tolerance to phytohormones, osmotic, and oxidative stresses (Figures 2, 4, 5; Supplementary Figure S2). Firstly, *TaHsfA2-13* was dramatically induced by heat stress (Figure 2B) and transgenic plants overexpressing *TaHsfA2-13* in Col-0 or *athsfa2* mutant showed improved thermotolerance (Figure 4). These results imply that the improvement of heat tolerance by *TaHsfA2-13* is conserved between monocot and dicot plant species. Next, we found that *TaHsfA2-13* was upregulated by other abiotic stresses besides heat stress, such as osmotic and oxidative stresses (Figure 2). Indeed, transgenic *Arabidopsis* lines clearly exhibited better growth status under H_2_O_2_ and mannitol treatments, with fewer withered leaves and higher chlorophyll content (Figure 5). Finally, we also found that *TaHsfA2-13* responds to SA, ABA and MeJA treatments (Figure 2). A large number of studies have shown that phytohormones play critical roles in helping plants to adapt to various environmental stresses (Verma et al., 2016). For instance, SA alleviates the adverse effects of heat stress on photosynthesis by altering proline production and ethylene formation (Khan et al., 2013). In general, ABA is responsible for plant defense against abiotic stresses such as drought and salinity (Zhang et al., 2006). MeJA operate in parallel, presumably synergistically, to ABA, and are indispensable for osmotic stress tolerance in rice (Tang et al., 2020). Therefore, our future research will focus on the potential molecular mechanism of TaHsfA2-13 regulated by phytohormones in response to various abiotic stresses.

Plant Hsps, as chaperone proteins, play key roles in biotic and abiotic stress tolerance of plants by maintaining the functional conformation of plant proteins and preventing the aggregation of non-native proteins (Ul Haq et al., 2019). Numerous studies have shown that the regulation of *hsps* by hsfs plays an important role in plant response to various environmental stresses (Guo et al., 2008; Xue et al., 2014; Driedonks et al., 2015). For example, the overexpression of *GmHsfA1* improved the thermotolerance of transgenic soybeans, possibly due to the activation of downstream genes *GmHsp70*, *GmHsp22* and other *GmHsps* (Zhu et al., 2009). In *Arabidopsis*, *Hsp90* is regulated by HsfA2 and enhances tolerance to oxidative stress (Nishizawa-Yokoi et al., 2010). During seedling stage of wheat, TaHsfA6e was reported to regulate the expression of *TaHsps* genes, such as *Hsp17*, *Hsp70* and *Hsp90* in response to high temperature and drought (Kumar et al., 2018). Additionally, *TaHsfA6f* could improve thermotolerance in wheat, which depended on the function of downstream target genes, including *TaHsps* (*TaHSP16.8*, *TaHSP17*, *TaHSP17.3* and *TaHSP90.1-A1*), *TaRof1*, *galactinol synthase*, and *glutathione-S-transferase* (Xue et al., 2015). In this study, with a few exceptions, almost all tested *Hsps* were strongly upregulated in *TaHsfA2-13* overexpressing transgenic lines after various stresses and phytohormones treatments (Figure 6). Previous studies have found that *HSPs* genes (*sHsp., Hsp70*, *Hsp90* and *DnaJ*) and Hsfs may be closely related to the structural integrity and function of chloroplast (Wu et al., 2018). Here, due to the protection of high level of Hsps, *TaHsfA2-13* overexpressing transgenic lines grew better and had higher chlorophyll content than WT (or *athsfa2*; Figures 4B, 5B; Supplementary Figure S2). Taken together, our results suggest that TaHsfA2-13 is a positive regulator of abiotic stresses, including heat, osmotic and oxidative stress, and involved in response to phytohormones treatments, possibly by upregulation of stress-related response genes, such as Hsps.

## Concluion

In summary, we clarified important roles of wheat *TaHsfA2-13* gene in response to HS, H_2_O_2_, mannitol, and SA in *Arabidopsis*. TaHsfA2-13 exerts stress tolerance functions by regulating the expression of downstream genes such as *Hsps*. It indicates that different signal pathways share the same signal molecules under stress conditions. Our study elevates the mechanistic understanding of individual gene involved in various stress and cross-talk tolerance in plant and provides genetic materials for crop stress tolerance breeding.

## Data Availability Statement

The datasets presented in this study can be found in online repositories. The names of the repository/repositories and accession number(s) can be found in the article/Supplementary Material.

## Author Contributions

XM, XG, and GL designed this study. XM performed bioinformatics analysis, generated and analyzed data, and wrote the manuscript. BZ, ML, RL, and QR performed the experiments. XG and GL edited this manuscript and contributed with valuable discussions. All authors contributed to the article and approved the submitted version.

## Funding

This work was supported by the Natural Science Foundation of Hebei Province (no. C2021301024); HAAFs Science and Technology Innovation Special Project (no. 2022KJCXZX-SSS-2); Talents Construction Project of Science and Technology Innovation of HAAFs (no. C22R1301); Basic Research Fund of Hebei Academy of Agriculture and Forestry Sciences (no. 2021110204).

## Conflict of Interest

The authors declare that the research was conducted in the absence of any commercial or financial relationships that could be construed as a potential conflict of interest.

## Publisher’s Note

All claims expressed in this article are solely those of the authors and do not necessarily represent those of their affiliated organizations, or those of the publisher, the editors and the reviewers. Any product that may be evaluated in this article, or claim that may be made by its manufacturer, is not guaranteed or endorsed by the publisher.

## Footnotes

^1^ www.lynnon.com

^2^ https://www.ncbi.nlm.nih.gov/

^3^ www.lynnon.com

## Supplementary Material

Supplementary Figure S1 Identification of transgenic plants. The mRNA levels of *TaHsfA2-13* overexpressing lines and WT (or *athsfa2*) were examined by semi-quantitative RT-PCR analysis, and the expression of *Atactin8* was analyzed as a loading control.

Supplementary Figure S2 Comparison of wild type (WT), *athsfa2* and *TaHsfA2-13* overexpressing transgenic plants in response to various abiotic stresses and phytohormones. **(A)** Morphology and **(B)** Chlorophyll content of WT, *athsfa2* and transgenic seedlings after various abiotic stresses and phytohormones. For each experiment, at least 30 plants per line were used. Values are means ± SD from three independent measurements. Different lowercase letters above the bars denote significant differences (*p* < 0.05).

## Abbreviations

Hsf: Heat shock transcription factor
HS: Heat shock
SA: Salicylic acid
WT: Wild type
Hsp: Heat shock protein
HSE: HS responsive element
DBD: DNA-binding domain
OD: Oligomerization domain
NLS: Nuclear localization signal
NES: Nuclear export signal
AHA: Activator peptide motif
