# Supplementary figures and images for: Characteristics and Regulating Roles of Wheat TaHsfA2-13 in Abiotic Stresses
Source: Front Plant Sci. 2022 Jun 27;13:922561. doi: 10.3389/fpls.2022.922561 (PMC9271894; doi:10.3389/fpls.2022.922561)

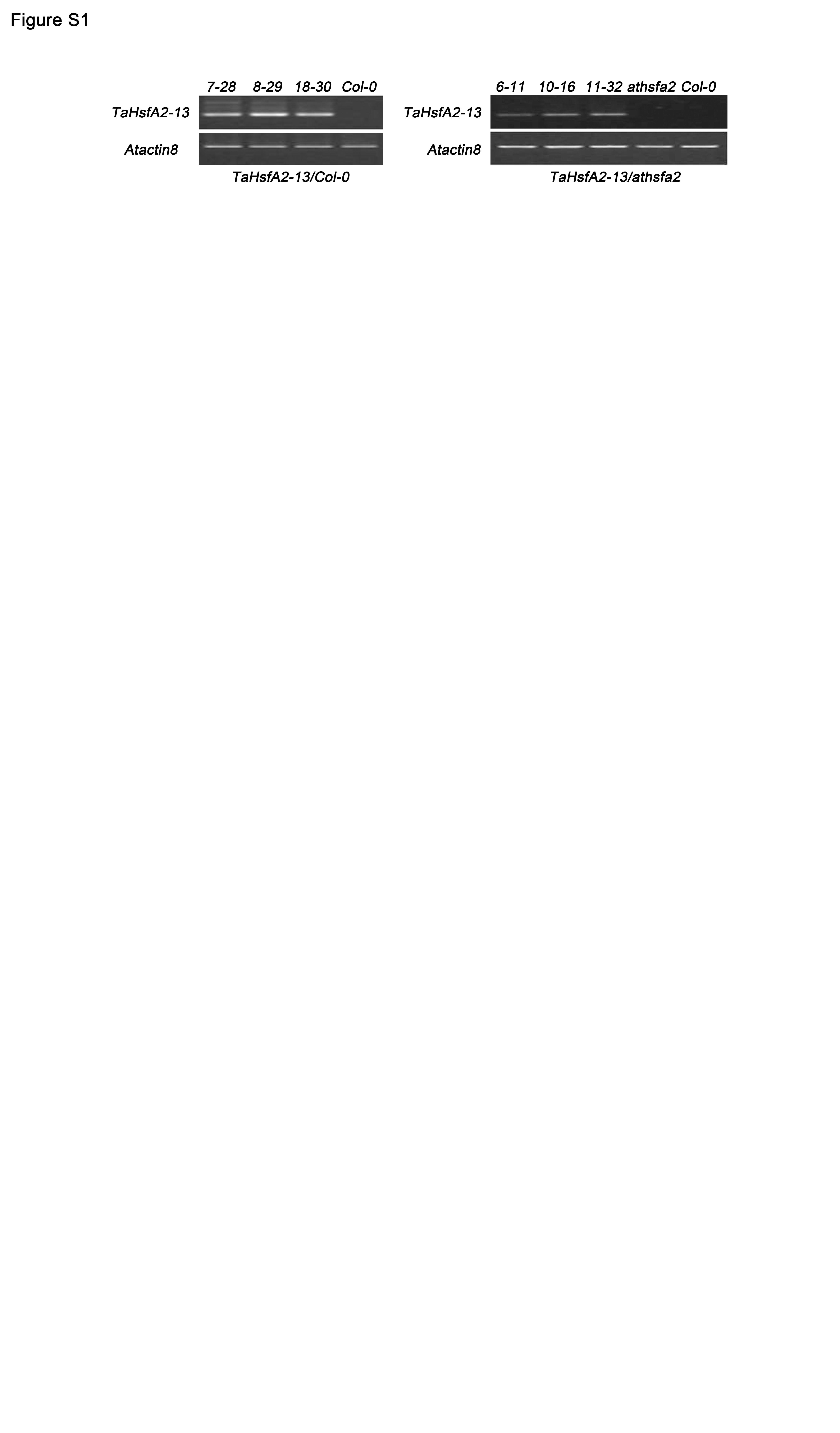

Supplement: Supplementary Figure S1 — Identification of transgenic plants. The mRNA levels of TaHsfA2-13 overexpressing lines and WT (or athsfa2) were examined by semi-quantitative RT-PCR analysis, and the expression of Atactin8 was analyzed as a loading control. [file Image_1.TIF]

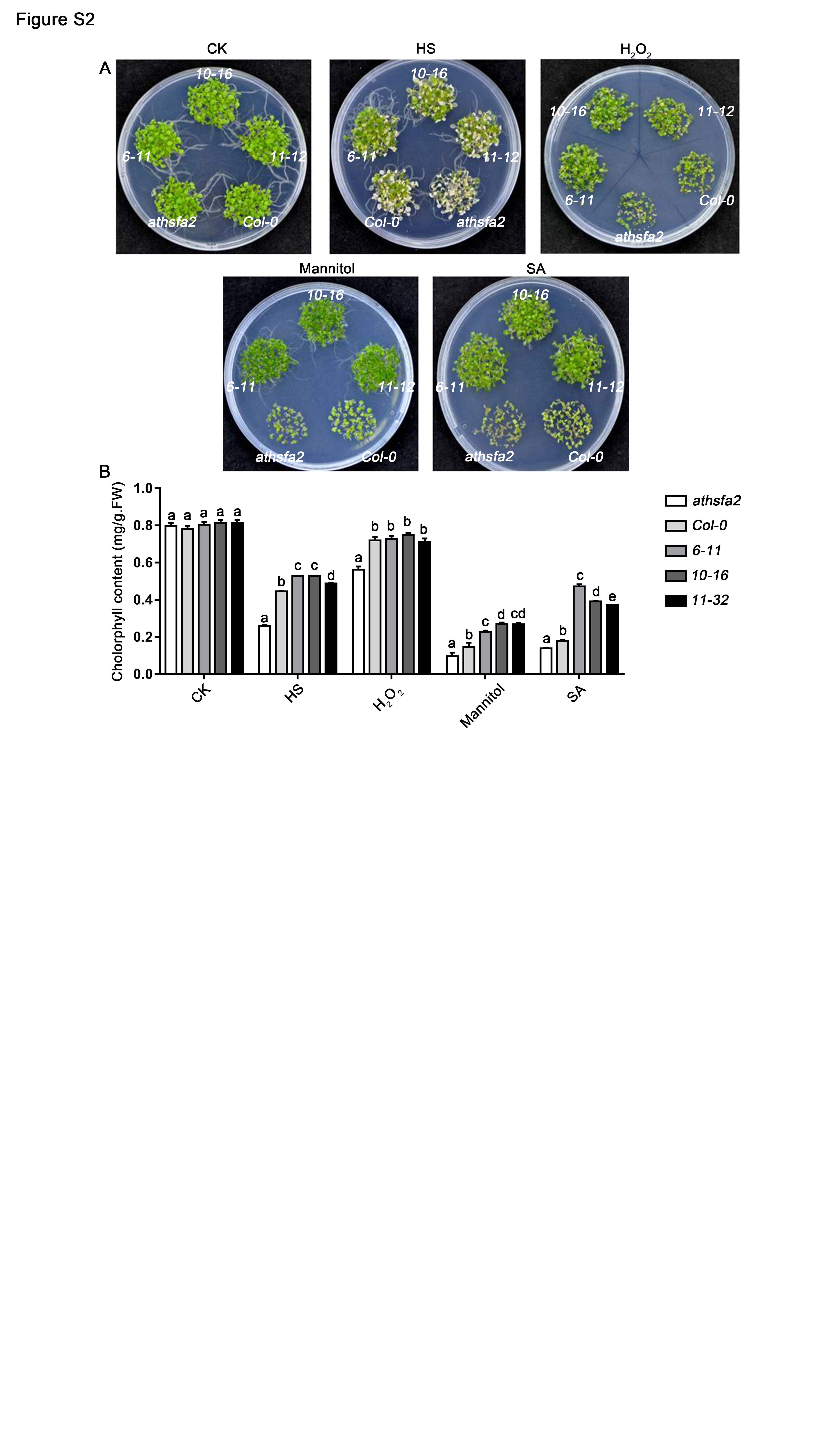

Supplement: Supplementary Figure S2 — Comparison of wild type (WT), athsfa2 and TaHsfA2-13 overexpressing transgenic plants in response to various abiotic stresses and phytohormones. (A) Morphology and (B) Chlorophyll content of WT, athsfa2 and transgenic seedlings after various abiotic stresses and phytohormones. For each experiment, at least 30 plants per line were used. Values are means ± SD from three independent measurements. Different lowercase letters above the bars denote significant differences (p < 0.05). [file Image_2.TIF]
