# Supplementary material for: Characteristics and Regulating Roles of Wheat TaHsfA2-13 in Abiotic Stresses
Source: Front Plant Sci. 2022 Jun 27;13:922561. doi: 10.3389/fpls.2022.922561 (PMC9271894; doi:10.3389/fpls.2022.922561)
Supplement: Supplementary file 3 [file Table_1.docx]

**TABLE S1 Primers used in this study.**

| Assays | Gene | Forward primer (5'→3') | Reverse primer (5'→3') |
| --- | --- | --- | --- |
| Gene Cloning | TaHsfA2-13 | ATGGACGCGATGCCGCCGGAGGGCATT | CTACTGCGGGCTAGTAGAACTCAGA |
| RT-qPCR | AtHsp21 | AAGTCCGCTACACCGTTCTC | CCAACAATCCGAAAGGAGAG |
|  | AtHsp70b | TCCGCTTAGCCTTGGACTT | ACGCCTGGTTGATTGTCTG |
|  | AtHsp70T | TGATTGAGGTGAGGATGCC | CCACTTCAACGACAAACCC |
|  | AtHsp90 | CCCTCTCTTCTTCATAAATCAACA | CCATCGCAACGAACTTTG |
|  | AtHsp101 | TGTCTTCAACACTCTGCTCCA | CACTTCCATTGTTACTTTCCCAG |
|  | AtActin8 | GCCAGATCTTCATCGTCGTG | TCTCCAGCGAATCCAGCCTT |
|  | TaRP15 | GCACACGTGCTTTGCAGATAAG | GCCCTCAAGCTCAACCATAACT |
|  | TaHsfA2-13 | TGAAGGAGGAGGAGGTGC | GTGTGGGTTACGCATTGC |
| Yeast One-Hybrid | AtHsp21 | GACTCACTATAGGGCGAATTCTTATTAGTTCATCAC | ATGCCAGGAATTACTAGTAGCCATTTGTTTCGA |
|  | AtHsp70b | GACTCACTATAGGGCGAATTCGGCTTTTCCTTTCTCGT | ATGCCAGGAATTACTAGTTGTTGCTAAAAAAAAGC |
|  | AtHsp70T | GACTCACTATAGGGCGAATTCGAAACTGAAAAAG | ATGCCAGGAATTACTAGTGAGCGTCAAAGC |
|  | AtHsp90 | GACTCACTATAGGGCGAATTCAGAAGACAAATGAGA | ATGCCAGGAATTACTAGTCTGAACATCCGCCATC |
|  | AtHsp101 | GACTCACTATAGGGCGAATTCCAATTAGCCAAGTTC | ATGCCAGGAATTACTAGTGATTATAGCGGTAATG |
|  | TaHsfA2-13 | GCCATGGAGGCCAGTGAATTCATGGACGCGATGCCG | CAGCTCGAGCTCGATGGATCCCTACTGCGGGCTAGT |
| Transactivation Analysis | TaHsfA2-13 | ATGGCCATGGAGGCCGAAATGGACGCGATGCCG | TGCGGCCGCTGCAGGTCGACCTACTGCGGGCTAGT |
|  | TaHsfA2-13^1-300^ | ATGGCCATGGAGGCCGAATTCATGGACGCGATGCCGCCGGAG | TGCGGCCGCTGCAGGTCGACCTACTCCTGAATGTTCTGCGCTAAATT |
|  | TaHsfA2-13^301-368^ | ATGGCCATGGAGGCCGAATTCATGCTTGGGCAGGGCAAAACAGAC | TGCGGCCGCTGCAGGTCGACCTACTGCGGGCTAGTAGAACTCAGATAC |
| Subcellular Localization | TaHsfA2-13 | GAGAACACGGGGGACTCTAGAATGGACGCGATGCCG | GCCCTTGCTCACCATGGATCCCTGCGGGCTAGTAGA |
| Genetic Transformation | TaHsfA2-13 | GAGAACACGGGGGACTCTAGAATGGACGCGATGCCG | CGATCGGGGAAATTCGAGCTCCTACTGCGGGCTAGT |
